# Sign and Speech Share Partially Overlapping Conceptual Representations

**DOI:** 10.1016/j.cub.2019.08.075

**Published:** 2019-11-04

**Authors:** Samuel Evans, Cathy J. Price, Jörn Diedrichsen, Eva Gutierrez-Sigut, Mairéad MacSweeney

**Affiliations:** 1 Institute of Cognitive Neuroscience, UCL, Alexandra House, 17-19 Queen Square, London WC1N 3AZ, UK; 2 Department of Psychology, University of Westminster, 115 New Cavendish Street, London W1W 6UW, UK; 3 Wellcome Centre for Human Neuroimaging, UCL, 12 Queen Square, London WC1N 3AR, UK; 4 Brain and Mind Institute, University of Western Ontario, London, ON N6A 3K7, Canada; 5 Deafness, Cognition and Language Research Centre (DCAL), 49 Gordon Square, London WC1H 0PD, UK

**Keywords:** speech, sign language, BSL, fMRI, semantics, bilingualism, conceptual representations, brain imaging

## Abstract

Conceptual knowledge is fundamental to human cognition. Yet, the extent to which it is influenced by language is unclear. Studies of semantic processing show that similar neural patterns are evoked by the same concepts presented in different modalities (e.g., spoken words and pictures or text) [1, 2, 3]. This suggests that conceptual representations are “modality independent.” However, an alternative possibility is that the similarity reflects retrieval of common spoken language representations. Indeed, in hearing spoken language users, text and spoken language are co-dependent [4, 5], and pictures are encoded via visual and verbal routes [6]. A parallel approach investigating semantic cognition shows that bilinguals activate similar patterns for the same words in their different languages [7, 8]. This suggests that conceptual representations are “language independent.” However, this has only been tested in spoken language bilinguals. If different languages evoke different conceptual representations, this should be most apparent comparing languages that differ greatly in structure. Hearing people with signing deaf parents are bilingual in sign and speech: languages conveyed in different modalities. Here, we test the influence of modality and bilingualism on conceptual representation by comparing semantic representations elicited by spoken British English and British Sign Language in hearing early, sign-speech bilinguals. We show that representations of semantic categories are shared for sign and speech, but not for individual spoken words and signs. This provides evidence for partially shared representations for sign and speech and shows that language acts as a subtle filter through which we understand and interact with the world.

## Highlights

• RSA analyses show that semantic categories are shared for sign and speech
• Neural patterns for individual spoken words and signs differ
• Spoken word and sign form representations are found in auditory and visual cortices
• Language acts as a subtle filter through which we interact with the world

## Teaser

Using fMRI, Evans et al. compare the similarity of neural patterns evoked by the same concepts presented as spoken British English and British Sign Language in hearing early, sign-speech bilinguals. Conceptual representations were only partially shared, suggesting that language acts as a filter through which we understand the world.

## Results

**Figure 1:**
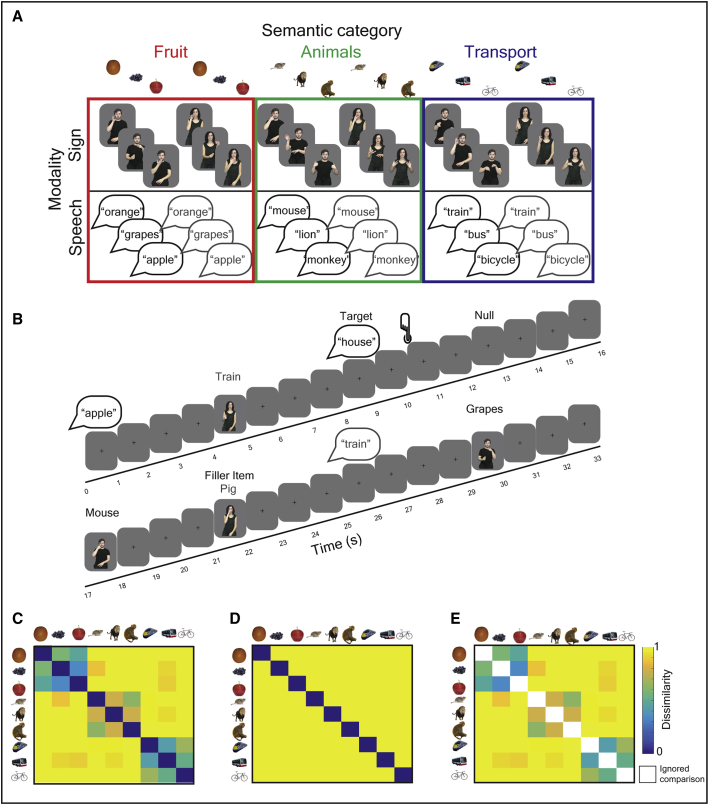
Stimuli, Experimental Design, and Semantic Models (A) Hearing, early sign-speech bilinguals were presented with 9 conceptual items that belonged to 3 semantic categories: fruit, animals, and transport. Items were presented as signs (videos) and spoken words (auditory presentation only) and were produced by male and female language models. (B) Within the scanner, participants attended to speech and sign and pressed a button to identify items that were not in one of the three target categories (e.g., house). (C–E) The dissimilarity between neural patterns evoked by the signs and spoken words were tau-a correlated with different theoretical models. The color bar reflects the degree of predicted semantic dissimilarity between items. (C) A semantic feature model derived from the Centre for Speech, Language and the Brain (CSLB) concept property norms [9]. (D and E) This model was decomposed into two independent components: (D) an item-based model that predicts that each item is uniquely represented, e.g., an “apple” is more dissimilar to other items than to itself and does not predict any broader semantic relatedness between items, and (E) a category-based model in which the between-item similarities are predicted by the semantic feature model but where the within-item similarities are not tested. White squares in this model indicate comparisons that were excluded.

Hearing early, sign-speech bilinguals were presented with 9 conceptual items from 3 semantic categories: fruit, animals, or transport, in a randomized event-related fMRI experiment. Each item was presented as a sign (video) or as a spoken word (audio only, not audio-visual) and was produced by a male or a female language model (Figure 1A). Participants were highly accurate (mean = 97%) at performing a within scanner semantic monitoring task (Figure 1B). Univariate general linear model (GLM) analyses indicated that speech and sign language engaged similar fronto-temporal networks, consistent with previous studies [10, 11, 12, 13, 14] (see Figure S2).

### Shared Semantic Representations for Speech and Sign

**Figure 2:**
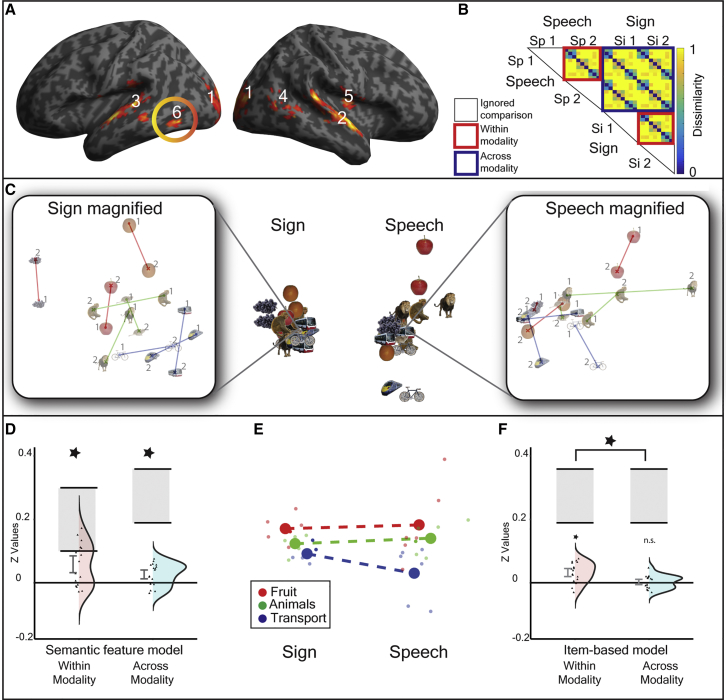
Shared Semantic Representations for Speech and Sign (A) A searchlight analysis identified brain regions containing positive within-modality representational distances, thresholded at p < 0.005 uncorrected at peak level, false discovery rate (FDR) corrected at q < 0.05 at the cluster level (extent threshold, k = 172 voxels). Clusters are numbered according to the text in the results section. Table S2 details the local maxima from this analysis. See Figure S2 for the univariate overlap between sign and speech, and see Figure S4 for tSNR maps showing how signal quality varied across the brain. (B) Representational distances in these regions were tau-a correlated with the semantic feature model within modality and across modality. The red boxes illustrate the within-modality distances, with the upper red box testing for abstracted speech representations (e.g., from speaker 1 to 2) and the lower red box testing for abstracted representations for sign (e.g., from signer 1 to 2). The blue box contains all across-language distances. Each 9 × 9 submatrix of dissimilarities is predicted from the semantic feature model (Figure 1C). White boxes are comparisons excluded from the analysis. The color bar reflects the predicted strength of dissimilarity. (C–F) Plots show the response in cluster 6, the left pMTG/ITG (−48 −62 −6). In this region, there was a fit to the semantic feature model within modality and across modality. However, when item-based and category-based representations were differentiated, this showed that the semantic category model (Figure 1E) was a fit within modality and across modality, but the item-based model (Figure 1D) was only a fit within modality. Further, the item-based model was a better fit within modality than across modality. (C) The non-metric MDS representation of the response in this region: the left panel shows within sign distances magnified to make the representational structure clearer, and the right panel shows the equivalent speech representations. In these magnified images, lines connect the same conceptual item produced by each speaker or signer, marked as speaker/signer 1 or speaker/signer 2 on the figure. (D) Plot shows the significant fit to the semantic feature model both within modality and across modality. Violin plots show distributions and individual data points for the z transformed values, including the 90% confidence interval and the noise ceiling (gray rectangle). (E) The non-metric MDS representation showing the mean centroid of each category within each modality for fruit (red), animals (green), and transport (blue), with dashed line connecting centroids across modality. Note the similar ordering of the category centroids both within and across each modality. (F) Plot shows the difference in fit to the item model within modality and across modality. See Figure S3 for the influence of sign iconicity on the left pMTG/ITG and Figure S1A for the definition of leave-one-out regions of interest (ROIs) for testing sensitivity to speaker and signer identity in this region.

Using a searchlight analysis, we first identified regions in which there were reliably positive representational distances (see STAR Methods) between items within modality (e.g., averaging speech-speech distances and sign-sign distances). We calculated distances only between items from the different language models (e.g., different speakers and signers, respectively) to exclude similarities driven by low-level perceptual properties. In these regions, we then tested for shared semantic representations using the following criteria: (1) a significant fit to the semantic feature model in the within-modality distances (i.e., speech-speech across speakers and sign-sign across signers; see Figure 2B, red boxes) and (2) a significant fit of the semantic feature model to the across-modality distances (i.e., speech-sign and sign-speech; see Figure 2B, blue boxes). We also expected (3) no evidence of a difference in strength of fit to the semantic model between speech and sign; (4) no evidence of low-level acoustic or visual sensitivity indicated by a fit to a model predicting greater distances between items from a different, as compared to the same, speaker in the speech-speech distances or from a different, as compared to the same, signer in the sign-sign distances; and (5) no fit to a model predicting sensitivity to the degree of iconicity of the signs, a perceptual feature present in sign, but not speech.

We found reliable within-modality distances in six clusters (Figure 2A): (1) in bilateral V1–V3 and the lateral occipital complex (LOC) (−14 −96 10); (2) the right anterior superior temporal gyrus (58 −4 −2); (3) the left anterior superior and middle temporal gyrus (−60 −10 −2); (4) the right middle temporal gyrus and middle temporal visual area (MT)/V5 (52 −68 6); (5) the right insular (36 −12 14); and (6) the left posterior middle and inferior temporal gyrus (left pMTG/ITG) (−48 −62 −6) (Figure 2; Table S2).

Three of these clusters showed a significant fit to the semantic model within modality (after adjusting alpha to p < 0.008 for six tests/clusters). These were found in the right middle temporal and V5/MT (cluster 4; t (16) = 3.946; p = 5.78 × 10^−4^; d_z_ = 0.957), the bilateral V1–V3 and LOC (cluster 1; t (16) = 3.837; p = 7.28 × 10^−4^; d_z_ = 0.931), and the left pMTG/ITG (cluster 6; t (16) = 3.622; p = 0.001; d_z_ = 0.879). However, the response in two of these clusters was not consistent with shared semantic representations because the fit to the semantic model was stronger for sign than for speech after adjusting alpha to p < 0.017 to account for 3 tests/clusters: right middle temporal and V5/MT cluster (t (16) = 2.842; p = 0.012; d_z_ = 0.689) and the bilateral V1–V3/LOC cluster (t (16) = 4.630; p = 2.78 × 10^−4^; d_z_ = 1.123). In both areas, there was a significant fit to the semantic feature model for sign (both p < 1.05 × 10^−4^), but not speech (both p > 0.110), and neither region showed a fit to the semantic model across modality (both p > 0.046).

**Figure 3:**
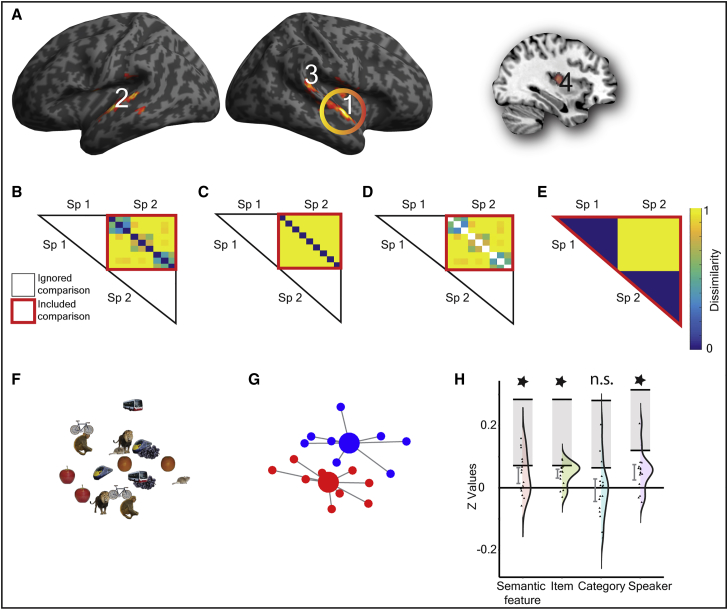
Speech-Specific Neural Responses (A) A searchlight analysis identified regions with greater representational distances for speech compared to sign, thresholded at p < 0.005 uncorrected at peak level, FDR corrected at q < 0.05 at the cluster level (extent threshold, k = 146 voxels). Clusters are numbered according to the text in the Results section. Table S2 details the local maxima from this analysis. (B–E) The within-speech models that were tested: (B) within-speech semantic feature model; (C) within-speech item-based model; (D) within-speech category-based model; and (E) between-speaker model. All models test dissimilarities across speaker (e.g., from speaker 1 to 2) in order to identify representations abstracted from perceptual features. Color bar reflects predicted strength of dissimilarity. White boxes are comparisons excluded from analysis. (F–H) The response in cluster 1, the right anterior STG (58 −4 −2), is shown. In this region, there was a significant fit to the semantic feature model, driven by item-based rather than category-based similarity and additional sensitivity to speaker identity. This is consistent with abstract spoken word form representations rather than modality-specific semantic processing. (F) The non-metric MDS solution. (G) Non-metric MDS highlighting speaker identity encoding in leave-one-participant-out ROIs (see Figure S1B). Large circles represent the centroids for items from speaker 1 (red) and speaker 2 (blue). Smaller circles represent the observed response for each item. Gray lines connect each item to centroid. (H) Violin plots show model fits for z transformed values for each model, with distributions and individual data points and 90% confidence intervals and noise ceiling (gray box shown).

**Figure 4:**
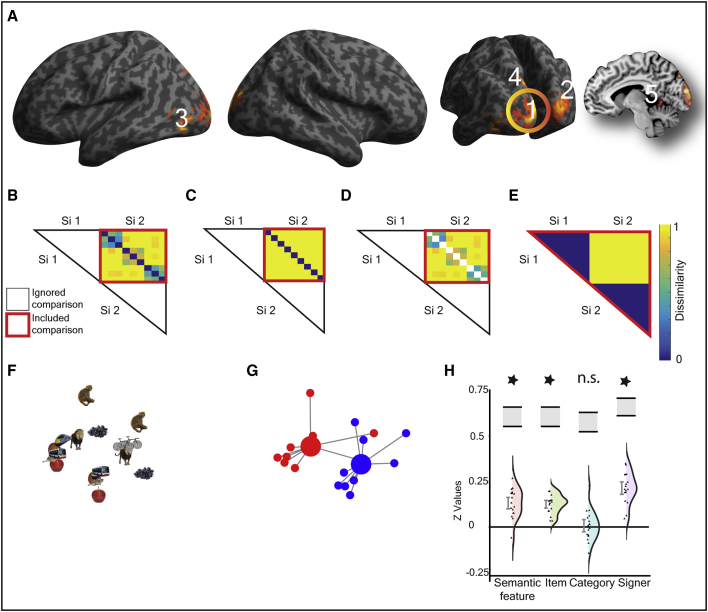
Sign-Specific Neural Responses (A) A searchlight analysis identified regions with greater representational distances for sign compared to speech, thresholded at p < 0.005 uncorrected at peak level, FDR corrected at q < 0.05 at the cluster level (extent threshold, k = 116 voxels). Clusters are numbered according to the text in the Results section. Table S2 details the local maxima from this analysis. (B–E) The within-sign models that were tested: (B) within-sign semantic feature model; (C) within-sign item-based model; (D) within-sign category-based model; and (E) between-signer model. All models test dissimilarities across signer (e.g., from signer 1 to 2) in order to identify representations abstracted from perceptual features. Color bar reflects predicted strength of dissimilarity. White boxes are comparisons excluded from analysis. (F–H) The response in cluster 1, the left V1–V3 (−6 −98 16), is shown. In this region, there was a significant fit to the semantic feature model, driven by item-based rather than category-based similarity structure and an additional sensitivity to signer identity, consistent with abstract sign form representations rather than modality-specific semantic processing. (F) The non-metric MDS solution. (G) Non-metric MDS highlighting signer identity encoding in leave-one-participant-out ROIs (see Figure S1C). Large circles represent the centroids for items from signer 1 (red) and signer 2 (blue). Smaller circles represent the observed response for each item. Gray lines connect each item to centroid. (H) Violin plots show model fits for z transformed values for each model fit, with distributions and individual data points and 90% confidence intervals and noise ceiling (gray box shown).

Only the response in the left pMTG/ITG was consistent with shared semantic representations (see Figure 2A, cluster 6). In addition to (1) fitting the within-modality semantic feature model (Figure 2D), the responses in this region showed (2) a significant fit to the across-modality semantic feature model (t (16) = 3.076; p = 0.004; d_z_ = 0.746; Figure 2D). There was also (3) no evidence for differential sensitivity in the encoding of semantics for speech and sign (t (16) = 0.400; p = 0.694; d_z_ = 0.097), (4) no sensitivity to the acoustic or visual features associated with speaker (see model in Figure 3E) or signer identity (see model in Figure 4E), both p > 0.060, and (5) no influence of the iconicity structure of sign in the sign-sign or across-modality distances, all p > 0.106 (Figure S3).

The fit of the semantic feature model (Figure 1C) can be decomposed into item-based dissimilarity (Figure 1D) and category-based dissimilarity (Figure 1E). For within-modality distances, the left pMTG/ITG showed a significant fit to both the semantic category (t (16) = 1.980; p = 0.033; d_z_ = 0.480) and item-based model (t (16) = 4.185; p = 3.50 × 10^−4^; d_z_ = 1.015). The critical analyses across modality indicated that the category-based model fit the data (t (16) = 2.509; p = 0.012; d_z_ = 0.608), but not the item-based model (t (16) = 0.475; p = 0.321; d_z_ = 0.115). There was no evidence of a difference in strength of fit to the category model within modality as compared to across modality (t (16) = 0.135; p = 0.894; d_z_ = 0.033), suggesting that semantic categories were represented robustly within and across modality. By contrast, the item model was a better fit to the within-modality than the across-modality distances (t (16) = 3.376; p = 0.004; d_z_ = 0.819; Figure 2F), showing that item-based representations are less robustly encoded across modality.

Taken together, the results suggest that semantic category structure drives similarity between sign and speech in left pMTG/ITG (see Figures 2C and 2E for the multidimensional scaling [MDS] solution, highlighting common category structure). As we did not observe the same effects in anterior temporal lobe (ATL) regions that have previously been associated with amodal semantic representations [15], we generated whole-brain temporal signal-to-noise ratio (tSNR) maps to compare signal quality across regions. These indicated that tSNR levels in the ATL were adequate and similar to the left pMTG/ITG (Figure S4).

### Modality-Specific Representations

In the absence of common category- and item-level representations, which would have been supportive of fully shared semantic representations, we tested for modality-specific semantic representations. Using a searchlight approach, we identified speech-specific and sign-specific regions by finding areas in which the average of the speech-speech distances were greater than the sign-sign distances and vice versa. In these regions, we tested for modality-specific semantic representations, evidenced by a significant fit to (1) the full semantic feature model (Figure 1C) and (2) to the semantic category model (Figure 1E) in the speech-speech or sign-sign distances for speech or sign, respectively, and (3) no evidence of a fit to the speaker or signer identity model (see models in Figures 3E and 4E) that would indicate a sensitivity to low-level visual or auditory features.

### Speech-Specific Responses

Four clusters showed greater representational distances for speech than sign: (1) right anterior superior temporal gyrus (STG) extending to the temporal pole (58 −4 −2); (2) left anterior STG (−56 −8 2); (3) right posterior STG/superior temporal sulcus (STS) (58 −34 18); and (4) right putamen and insula (30 −10 10) (Figure 3A; Table S2). None of the regions showed speech-specific semantic representations, as the category-based model (Figure 3D) was not a significant fit (all p > 0.110) after adjusting alpha to p < 0.013 to account for four clusters/tests. In one of the clusters, the right anterior STG (58 −4 −2) (Figure 3A, cluster 1), there was a significant fit to the semantic feature model (t (16) = 2.529; p = 0.011; d_z_ = 0.613; Figures 3B and 3H). However, this was driven by a fit to the item-level model (t (16) = 5.229; p = 4.14 × 10^−5^; d_z_ = 1.268; Figures 3C and 3H) and was accompanied by sensitivity to the acoustic differences between speakers (t (16) = 3.325; p = 0.002; d_z_ = 0.806; Figures 3E and 3H). This pattern of response is consistent with speech form representations rather than speech-selective semantic representations (Figures 3F and 3G for MDS solution highlighting speaker-based similarity). Identification of spoken word forms in the right anterior STG was unexpected. This may reflect the greater involvement of the right hemisphere in language processing in early bilinguals [16] or, given the reported greater importance of the right hemisphere in sign processing in hearing native signers [17], may reflect an effect more specific to early sign-speech bilinguals.

### Sign-Specific Responses

Five regions showed greater representational distances for sign than speech: (1) a cluster spreading across left V1–V3 (−6 −98 16); (2) a cluster within right V1–V3 (22 −90 16); (3) a cluster in the left LOC and MT/V5 (−44 −80 −6); (4) left superior occipital gyrus and superior parietal lobule (−10 −84 42); and (5) left lingual gyrus spreading to the cerebellum (−4 −48 −8) (Figure 4A; Table S2). Activity in these regions was not consistent with sign-specific semantic representations, as the category-based model was not a significant fit in any region (all p > 0.037) after adjusting alpha to p < 0.010 for five clusters/tests. The response in the clusters in the left V1–V3 and right V1–V3 were analogous to those for speech. Activity patterns were characterized by a fit to the semantic feature model (both p < 3.10 × 10^−5^) but driven by item-based encoding (p < 1.34 × 10^−7^) with additional sensitivity to signer identity (both p < 3.07 × 10^−6^; Figure 4), consistent with sign form representations.

## Discussion

Our findings indicate that semantic representations for sign and speech are shared but only at a broad level of semantic specificity. In the left pMTG/ITG, both individual items and categories were encoded within modality, but across modality, this was true only for categories. Moreover, item-level encoding was significantly stronger within as compared to across modality. In sign-specific and speech-specific regions, we found item-based rather than category-based coding. These representations retained sensitivity to auditory and visual features, suggestive of phonological word and sign form representations rather than language-specific semantic representations.

Shared category representations for sign and speech in left pMTG/ITG are consistent with studies showing common categories for items presented as pictures, environmental sounds, and speech and text within this region [1, 2]. Indeed, activation of the left pMTG/ITG is associated with extraction of meaning from both sound and vision. It is activated when reading words [18], perceiving semantically ambiguous speech [19] and sign language [20, 21, 22]. However, the loci of shared representation is more posterior than the more anterior temporal lobe regions associated with amodal semantics predicted by the “hub and spokes” model of semantic cognition [15]. Plausibly, the more posterior convergence identified in our study may be influenced by visually derived language representations of sign that may be found closer to the primary visual cortices. In contrast, amodal processing in ATL has been observed in studies of spoken language, either in healthy individuals or those with semantic dementia. Users of only spoken languages do not have visually derived language representations in the same way that signers do. We learn to read alphabetic scripts by making strong associations between orthography and speech sounds [4]. Similarly, pictures likely activate dual visual-verbal processing routes in spoken language users [6]. Our work highlights the unique contribution that sign languages provide in understanding semantic cognition. Future studies with healthy sign language users, deaf and hearing, and those with semantic dementia will contribute toward more complete models of semantic processing.

Common semantic coding was limited to category- and not item-level representations. This subtle divergence between languages is consistent with the notion that language influences, rather than determines, perception and thought [23, 24]. These data make a novel contribution, because we compared neural responses to languages that differ substantially in their linguistic structure, using sensitive multivariate statistical methods. However, we do not claim that our findings are necessarily specific to the contrast between signed and spoken languages. Our results are consistent with previous work that failed to show cross-decoding between individual spoken and written words across languages in English-French bilinguals [25], although that study did not test for category coding. Further work should investigate whether similar mechanisms underlie both findings. Studies testing for item- and category-based similarity for text, speech, and sign in sign-speech bilinguals and between stimuli in different modalities in spoken language bilinguals using typologically close and distant spoken languages will clarify the specificity of our findings. Contrasts of representations of signs in deaf signers and speech in hearing monolinguals will further clarify the influence of language experience on such representations.

Why are conceptual representations shared at only a coarse level of semantic specificity? Partially shared semantic representations between languages are consistent with computational models of bilingualism, such as the distributed feature model [26]. These models predict a single semantic store, in which each language weights semantic features independently [26, 27, 28]. One factor contributing to differing weights between sign and speech may be the greater polysemy (lexical items having more than one meaning) exhibited in signed languages [29]. Another may be a consequence of differences in phonology. Studies of spoken language show that lexical-semantic access is affected by the phonological structure of the lexicon. Words from dense phonological neighborhoods activate semantic representations less strongly [30] due to cascading activation between phonology and semantics [31]. Signed and spoken languages have very different phonologies and therefore phonological neighborhoods. This might affect the strength and structure of semantic activation within sign and speech lexicons, reducing the commonality of conceptual representations between the languages.

Another explanation is that the greater iconicity found in sign languages [32] reduces the degree of similarity between sign and speech. Although we did not observe an effect of iconicity in the response in the left pMTG/ITG, which would have directly supported this explanation, there are also more opaque form-meaning links that differ across speech and sign. For example, the handshape “I” (extension of the little finger alone) denotes a number of British Sign Language (BSL) signs that have negative connotations: bad; wrong; and poison [33]. Similarly, English words beginning with “gl” are often associated with light of low intensity: glow; glint; and glimmer [32]. Some canonical signs also carry additional layers of meaning that communicate size, location, movement, and other features of the referent: aspects of meaning that cannot be communicated by the voice. These features may fundamentally differentiate semantic representations for sign and speech. Given this, we might predict differences in the representation of specific semantic categories. For example, representations for tools might be expected to differ between unimodal (e.g., speech-speech) and bimodal (e.g., sign-speech) bilinguals on the basis that signs for objects would evoke greater specificity in the semantic features associated with how they are handled, particularly in sign languages that emphasize the handling properties of objects [34].

To conclude, our results suggest that the language that we use to communicate acts as a subtle filter through which we understand and interact with the world. This finding is unexpected. Previous brain-imaging studies showing significant univariate overlap of activation for sign and speech [10, 11, 12, 13, 14] have led researchers, including ourselves, to propose extensive similarity in the neural processes underlying sign and speech [35]. Our findings suggest the need to rethink this assumption and highlight the unique perspective that sign language can provide on language processing and semantic representation more broadly.

## STAR★Methods

### Key Resources Table

**Table:** 

REAGENT or RESOURCE	SOURCE	IDENTIFIER
**Deposited Data**		

Raw fMRI data	This paper	Available on request
Stimulus materials: sign and speech samples	This paper; Mendeley Data	Mendeley data (https://doi.org/10.17632/3d983g83v5.1)
Thresholded Group level RSA Searchlight maps	This paper; Mendeley Data	Mendeley data (https://doi.org/10.17632/3d983g83v5.1)
Group level summary data for Region of Interest (ROI) Analyses	This paper Mendeley Data	Mendeley data (https://doi.org/10.17632/3d983g83v5.1)

**Software and Algorithms**		

MATLAB 2018a	The mathworks	RRID:SCR_001622
RSA toolbox	[36]	https://github.com/rsagroup/rsatoolbox

### Lead Contact and Materials Availability

Further information and requests for resources and reagents should be directed to and will be fulfilled by the Lead Contact, Dr Samuel Evans (S.Evans1@westminster.ac.uk). All materials are available upon request.

### Experimental Model and Subject Details

#### Participants

Ethical approval was granted by the UCL ethics committee and informed consent was obtained from all participants. Data were collected from 18 right handed early sign-speech bilinguals with no known neurological, hearing or language learning impairments. One participant’s data was removed from the set due to an incidental finding, leaving a final dataset of 17 participants (Mean age = 33; range 20-52 years; female = 12). All of the participants were born and educated in the UK, except for one who was born in Australia and another who was born in a non-English speaking country, but moved to the UK at the age of three. Fifteen participants learned British Sign Language (BSL) from a deaf parent and two from an older deaf sibling. Two of the participants who learned sign language from a deaf parent did not learn BSL from birth; one, learned AUSLAN from birth and learned BSL from the age of twenty-one, the other, was exposed to another sign language from birth, before learning BSL from 3 years of age. Participants judged themselves to have excellent BSL skills on a self-report scale (1 poor - 7 excellent): mean = 6.3/7, SD = 0.86, range = 4-7. Six participants had previously worked as a BSL interpreter or were currently training to be an interpreter. One was a BSL teacher and three had worked or were working as Communication Support Workers (CSWs). All participants reported having previously interpreted in an informal capacity for a family member.

### Method Details

#### Speech and sign stimuli

Stimuli consisted of nine core items for which neural responses were analyzed. Each core item was presented 48 times across the whole experiment, in different modalities (sign/ speech) and by different models (male/ female) (see ‘fMRI paradigm’ for more details). These nine items belonged to three categories: fruit (orange, grapes and apple), animals (mouse, lion and monkey) and transport (train, bus and bicycle). Items within each category were similar and were distinct from other categories on the basis of their semantic features, as evidenced by the CSLB concept property norms [9] (see Figure 1C). Items were chosen to ensure that the categories were matched for age of acquisition (fruit M = 3.78; animals M = 4.52; transport = 4.04), imageability (fruit M = 618; animals M = 610; transport M = 622), familiarity (fruit M = 566; animals M = 521; transport M = 551) and the number of syllables and phonemes in spoken English [37, 38, 39] (see Table S1 for full details). In addition, we ensured that the BSL equivalents of the spoken words were matched across category for handshape, location, movement and handedness, and that iconicity [40] was similar across categories (fruit M = 3.80; animals M = 3.92; transport M = 4.23; 1 low - 7 high iconicity). Iconicity ratings from the participants’ were significantly correlated with those collected from deaf BSL users by Vinson et. al. [40] (n = 18, r = 0.917, p = 2.22 × 10^−07^).

Speech samples were recorded by a male and female Southern British English (SBE) speaker in an acoustically shielded booth with 16-bit quantisation and a sampling rate of 22050 Hz using Adobe Audition. These were auditory only, rather than the auditory-visual presentations typically used in studies comparing speech and sign language processing [20]. Auditory only speech presentations ensured that speech and sign were maximally different from each other and that any observed commonalities could not be attributed to common visual features. Auditory recordings were excised at the zero crossing point. They were then filtered to account for the frequency response of the Sensimetric headphones used in the scanner (http://www.sens.com/products/model-s14/) and the overall amplitude was Root Mean Square (RMS) equalised to ensure a similar perceived loudness. The mean duration of the auditory stimuli for the core items was 558ms (range = 323-865 ms), these sounds were similar in duration across semantic categories (fruit M = 573 ms; animals M = 575 ms; transport M = 533 ms) and gender of the speaker (male M = 557 ms; female M = 564 ms). The phonological distance between each of the spoken words was calculated using the Levenshtein distance [41]. This was achieved by calculating the number of phoneme insertions, deletions and/or substitutions necessary to turn one word into the other, divided by the number of phonemes in the longest word. The absolute value of the difference in Levenshtein distance between each item was calculated. These distances did not correlate with the semantic feature distances (r = 0.063, n = 36, p = 0.713), hence semantic structure was not confounded with phonemic structure.

The BSL signs were all common variants in southern England as shown in the BSL SignBank [42] (https://bslsignbank.ucl.ac.uk/dictionary/). Signs were recorded with a Sony Handycam HDR-CX130 on a blue background by a male and a female deaf native signer with a sampling rate of 50 fps and an aspect ratio of 1920x1080. The blue background was keyed out and replaced with a dark gray background. Videos were down-sampled to 30 frames per second and a resolution of 960 × 540 with Adobe Premiere for presentation in the scanner. All signs were produced with corresponding BSL mouthing. The signs were recorded in isolation such that the hands returned to a neutral position resting on the knees between each sign. During editing, the start and end-points of a sign were identified as a ‘hold’ (very brief pause in movement of the hands) to remove the transitional movement into and out of the neutral hands on the lap. Still frames of the hold points at the beginning and end of each sign, with duration of 333ms, were inserted to ensure that the signs were easily perceived in the scanner. The mean duration of the sign stimuli was 1107ms (range = 867-1400ms). The signs were similar in duration as a function of semantic category (fruit M = 1079ms; animals M = 1055ms; transport M = 1128ms) and gender of the signer (male M = 1087ms; female M = 1086ms).

An iconicity dissimilarity measure [40] for the signs was calculated by taking the absolute value of the difference between ratings of each item with every other. These distances did not correlate with semantic feature similarity (r = −0.126, n = 36, p = 0.465), hence semantic structure was not confounded with iconicity.

Participants were shown 36 additional items in the scanner to facilitate a semantic monitoring task (see Figure 1B) for which neural activity was not analyzed. The additional items consisted of 18 items from outside the categories of fruit, animal and transport, e.g., buildings, clothes, furniture and tools, which were included as target filler trials. Plus, an additional 18 non-target filler trials, 6 per category, of other types of fruit, animals or transport that were included to reduce habituation to the nine core items (see ‘fMRI Paradigm’ below for details of number of presentations). Each individual filler item was produced by only one of the speakers or signers, with the number of items from each speaker and signer balanced.

Prior to scanning, participants were familiarised with the signs and spoken words used in the study. Participants saw each sign stimulus produced by both sign models and were required to translate the word into spoken English. They also heard each word produced by both speech models and were required to repeat the spoken word aloud. They were shown all core items, target and non-target fillers. Sign recognition was high (core items: mean = 17/18, min = 15/18, max = 18/18; filler items: mean = 32/36, min = 21/36, max = 35/36). On very few occasions participants interpreted a sign as a non-intended English word. Typically when this occurred participants provided a translation that reflected their regional variant of BSL. When participants were asked if they knew any other meanings of the sign, they were usually able to provide the target translation. They were then asked to interpret the sign, on this occasion, as the target translation for the study. They were then retested on all the items in the experiment to ensure retention. Seventeen out of 18 participants required one round of correction, the remaining participant required a second round. Participants practiced a mock version of the within scanner task on a laptop prior to scanning.

#### fMRI task

In the scanner, participants were required to attend to the signed and spoken stimuli and to press a button when they encountered an item from outside the categories of fruit, animals or transport, e.g., a target filler item (see Figure 1B). The handedness of the button press was counterbalanced across participants. On average 97% of outside category target items were identified (mean 35/36 correct, SD = 1.45, min = 31, max = 36) and accuracy was significantly greater than chance (mean d’ score = 4.56), t (16) = 42.74, p = 6.37 × 10^−18^, indicating that participants were fully engaged with the task.

Data were collected in 6 runs. In each run, each of the 9 core items were presented twice in each of the following formats: sign and speech; male and female model. Therefore, each core item was presented 8 times in each run (2x2x2), with 72 core trials in total (9 items x 8 instances). Within each run, core items were presented as two concatenated mini blocks of 36 trials. Within each mini block items were randomized with the constraint that the same concept (e.g., ‘orange’) could not be presented consecutively, regardless of modality, to reduce habituation.

In addition, in each run there were 6 target filler trials (non-fruits, transport or animals) for which participants were required to press a button and 6 non-target fillers (‘other’ fruits, transport or animal items). The total number of trials was balanced within run for modality (e.g., whether sign or speech) and language model (e.g., speaker and signer). The filler trials (target and non-target fillers) were interspersed within each run regularly but unpredictably. An additional, seven null trials lasting 4 s were regularly but unpredictably interspersed within the each run. During these trials a white fixation cross was presented on a gray background in the absence of sound or additional visual stimulation for 4 s.

In summary, each of 6 runs consisted of 91 trials (72 core trials, 6 target filler trials, 6 non-target filler trials, 7 null trials). The order of modality of presentation of the items (speech/sign) was counter balanced across pairs of participants, such that items presented as signs to participant 1 were presented as speech to participant 2, and vice versa. Each stimulus was presented for its natural duration and was followed by a fixation cross lasting 3 s, before the start of the next trial.

After scanning, participants provided iconicity ratings on the sign stimuli that they had viewed in the scanner using the technique described by Vinson et al. [40]. They then took part in a multiple arrangement task in which they arranged pictures of the core and filler items “based on their similarity” using a drag and drop interface [43]. The Euclidean distances derived from this arrangement correlated highly with the CSLB concept property norms for the core items (r = 0.904, n = 36, p = 4.42 × 10^−14^), suggesting that the semantic feature norms provided a good summary of the semantic space of our participant group.

#### MRI Data Acquisition

Data was acquired with a 3-Tesla scanner using a Magnetom TIM Trio systems (Siemens Healthcare, Erlangen, Germany) with a 32 channel headcoil. A 2D epi sequence was used comprising forty 3mm thick slices using a continuous ascending sequence (TR = 2800ms, TA = 2800ms, FA = 90°, TE = 30ms, matrix size = 64x64, in-plane resolution: 3mm x 3mm, interslice gap = 1mm). Six runs of data were acquired each lasting ∼6-7 minutes with around 136 brain volumes collected per run; the exact number of volumes was dependent on the stimuli included in each run. EPI data collection lasted around 45 minutes. This was followed by a fieldmap, acquired using a double-echo FLASH gradient echo sixty-four slice sequence (TE1 = 10ms, TE2 = 12.46ms, in-plane view 192x192 mm, in-plane resolution: 3mm x 3mm, interslice gap = 1mm). At the end of the session a high-resolution T1 weighted structural image was collected using a 3D Modified Driven Equilibrium Fourier Transform (MDEFT) sequence (TR = 1393ms, TE = 2.48ms, FA = 16°, 176 slices, voxel size = 1 × 1 × 1 mm).

In the scanner, stimuli were presented using the COGENT toolbox (http://www.vislab.ucl.ac.uk/cogent.php) running in MATLAB. Auditory stimuli were presented at the same comfortable listening level for all participants. Visual images were presented using a JVC DLA-SX21 projector, with a screen resolution of 1024x768 and frame rate of 60Hz, using back projection onto a within bore screen at a distance of 62cm from the participants’ eyes.

### Quantification and Statistical Analysis

#### Univariate Analysis

Data were analyzed using SPM12 (https://www.fil.ion.ucl.ac.uk/spm/) using MATLAB. The first six images of each run were removed to account for T1 equilibrium effects. The structural and functional images were centered at the anterior commissure. Functional scans were slice time corrected to the middle slice, realigned to the first image and unwarped using field maps. The structural image was co-registered to the mean functional image. The parameters derived from segmentation, using the revised SPM12 segmentation routines, were applied to normalize the functional images that were re-sampled to 2x2x2mm. The normalized images were then smoothed with a Gaussian kernel of 6-mm full-width half maximum. Data were analyzed using a general linear model with a 360 s high-pass filter and AR1 correction for auto-correlation. In the first level design matrices, events were modeled with a canonical hemodynamic response function marking the onset of the stimulus and duration in seconds. The design matrices included a regressor for the onset of the speech trials, sign trials, filler target and non-target trials in each modality (4 regressors), button presses when the target was present in each modality (e.g., hits) (2 regressors) and button presses when the target trials were absent for each modality (e.g., false alarms) (2 regressors), six movement regressors of no interest and the session means. The rest condition constituted an implicit baseline. Contrast images of [speech > rest] and [sign > rest] were taken to the second level to conduct one sample t tests.

#### Representational similarity analysis (RSA)

At the first level, data were analyzed with SPM12. Analyses were conducted in native space. Images were slice time corrected to the middle slice, realigned to the first image and unwarped using fieldmaps, but were not normalized or smoothed. The images were segmented, using the revised SPM12 segmentation routine, to estimate the transformation from native space to MNI space and vice versa. In the first level model in native space, the two repetitions of each core item presented in each modality and by each speaker and signer were modeled as a separate regressor (36 regressors: 9 core items x 2 modalities x 2 language models). Additional regressors were included modeling the onset of filler target and filler non-target trials for each modality (4 regressors), plus button presses when the target was present in each modality (e.g., hits) (2 regressors) and button presses when the target trials were absent for each modality (e.g., false alarms) (2 regressors). This constituted 42 regressors per run, plus 6 motion parameter regressors and 6 session means. A high pass filter set at 360 s and AR(1) correction was applied. RSA analysis was conducted with the latest version of the RSA toolbox (https://github.com/rsagroup/rsatoolbox) [36]. The representational distances estimated from the first level betas were used to calculate the cross-validated Mahalanobis (crossnobis) distances using the RSA toolbox [36]. These crossnobis distances employ multivariate noise normalization that down-weight correlated noise across voxels, thereby increasing sensitivity to experimental effects [44]. The cross-validation across imaging runs ensures that the estimated distances between neural patterns are not systematically biased by run-specific noise, which allows us to test the distances directly against zero (as one would test cross-validated classification accuracy against chance). Therefore, the crossnobis distance provides a measurement on a ratio scale with an interpretable zero value that reflects an absence of distance between items.

#### Searchlight RSA analyses

A volumetric searchlight analysis [45] was conducted using a spherical 8mm searchlight containing 65 voxels, consistent with the parameters used in previous studies of language processing [46]. In the searchlight analysis, the crossnobis distance between each core stimulus and every other was calculated to generate a Representational Dissimilarity Matrix (RDM) for every voxel and its surrounding neighborhood. The resulting RDM reflected sign-sign, speech-speech or speech-sign distances, that constitute within and across-modality dissimilarities. In the searchlight analyses, the average of speech-speech and sign-sign distances (e.g., combined within-modality distances) and the average of the speech-speech and sign-sign distances separately were returned to the voxel at the center of each sphere in three separate searchlight analyses. Within-modality distances were calculated only between items from the different language models (e.g., different speakers and signers respectively) to exclude similarities driven by low-level perceptual properties. Each participants’ native space whole brain searchlight map was normalized to MNI space. These maps were inclusively masked with a > 20% probability gray matter mask, using the canonical MNI brain packaged with SPM12. The resulting normalized, masked images were submitted to SPM12 for one sample t tests testing for greater than zero within-modality distances and paired t tests testing for differences between the speech-speech and sign-sign distances at the second level. All statistical maps are presented at an uncorrected peak level threshold of p < 0.005, FDR cluster corrected at q < 0.05 to identify regions of interest for subsequent analysis. Extent thresholds were as follows: within-modality distances (k = 172 voxels), speech > sign distances (k = 146 voxels) and sign > speech distances (k = 116 voxels).

#### Regions of Interest (ROI) Analyses

The clusters identified from the searchlight analyses were used as Regions of Interest (ROIs) in which to test theoretical models of brain function. Note that ROI analyses are advised when testing special populations in which sample sizes are necessarily restricted [47]. Using ROIs that contain reliable representational structure, e.g., greater than zero distances, provides an additional protection against spurious distance-model correlations in regions in which there is no reliable representational structure. This approach is an efficient and statistically powerful way to generate ROIs as it uses all the data [48].

As each cluster contains multiple RDMs, one for each searchlight contained within the cluster, the RDMs were averaged, to provide a single representative RDM for each cluster, and each participant. These distances were then used to test hypothetical models of brain function (described below). The non-parametric Tau-a correlation was used in preference to Pearson or Spearman correlation as the models contained tied ranks [36]. The resulting correlation coefficient was converted to a Pearson’s r value, then to a Fisher-transformed Z value, to permit parametric statistical analysis [49]. Noise ceilings [36] were estimated within-modality and across-modality separately as appropriate for each model. The lower bound was estimated by calculating the mean z converted Tau-a correlation coefficient between each participant’s RDM and the average RDM for the group excluding that participant (e.g., leaving one participant out). This is an estimate of the fit that should be achieved if the theoretical model captures all systematic variation in the RDM across subjects in this region. The upper bound was estimated by calculating the mean z converted, Tau-a correlation between each participant’s RDM and the average RDM for the group including that participant. This value constitutes a theoretical maximum of the best possible fit that can be achieved between the data and a model with this region. These limits provide a benchmark against which to assess the quality of model fit as they reflect the bounds of the best possible model fit that could be expected given the noise in the data.

#### RSA Models

A semantic model was tested using the CSLB concept property norms [9] (Figure 1C). This kind of feature-based semantic model can account for the ability to categorize by semantic group, e.g., a zebra is an animal, and to tell-apart unique items, e.g., that a zebra differs from a horse. As such, the similarities expressed by the model can be decomposed into two independent components. One, an item-based model that predicts that each item is uniquely represented, e.g., an ‘orange’ is more dissimilar to all other items than to itself, and does not predict any other relatedness between items (Figure 1D). The other, a model in which item-to-item similarities are not tested, but category structure is predicted (Figure 1E) – referred to as a category-based model. An additional model testing for dissimilarities based on speaker (Figure 3E) and signer identity (Figure 4E) was also tested, e.g., models predicting trials from speaker/signer 1 to be more dissimilar than trials from speaker/signer 2, and vice versa. The purpose of this model was to test for neural dissimilarities based on lower level acoustic and visual features.

These models can be tested within-modality, e.g., correlated within speech-speech and sign-sign distances combined or separately, or across-modality, e.g., correlated with speech-sign distances. The testing of models using across-modality distances is equivalent to cross decoding representational structure between speech and sign, positive evidence provides support for common representational structure across languages [50]. Note that we only test for across-modality semantic representations in areas in which there is evidence of within-modality representational structure. As negative correlations are not plausible, greater than 0 model fits were assessed with one-tailed, one sample t tests. Two-tailed paired t tests were used to assess differences in fit between models. Multidimensional Scaling (MDS) was conducted to visualize the similarity structure of the RDMs by calculating the averaged participant RDM and applying non-metric MDS, consistent with the non-parametric correlational approach.

It is important that the RSA models were evaluated within regions of interest that were defined in a manner that is statistically unbiased [51]. We tested RSA models in regions identified as having positive within modality distances or larger relative distances for speech than sign, and vice versa. The between speaker and/or between signer distances were used to define ROIs. Analyses that evaluate models that use only the between speaker and signer distances are orthogonal to ROI selection. This is because the mean distance is implicitly subtracted out in the correlation between the model and the distances [52]. This is true of all the models tested in this study except the speaker and signer identity models. These models predict larger distances for the between speaker/signer than the within speaker/signer distances. As the ROIs are defined on the basis that they show non-zero across speaker/signer distances, the testing of these models would not be orthogonal to ROI selection. Therefore, for these models, to ensure that ROI selection was orthogonal, we generated leave-one-participant-out ROIs to evaluate the fit of the speech and signer identity models [53]. That is, to identify an ROI for Participant 1, we re-estimated the random effects t test using the whole-brain searchlight maps for the within modality, speech > sign and sign > speech distances, with Participants 2 to 17, and so forth for all participants. We thresholded these maps at p < 0.001 (uncorrected) to extract the clusters. This threshold identified discrete clusters, in the same regions as the full group model in all leave-one-out permutations. This generated 17 subtly different ROIs, that were statistically independent, which were used to evaluate the model fit of the speaker/signer identity models (see Figure S1 for the location and overlap between these ROIs).

### Data and Code Availability

Anonymised group level data and stimulus materials are available at Mendeley Data (https://doi.org/10.17632/3d983g83v5.1). The raw MRI data supporting the current study have not been deposited in a public repository, as the participants did not consent to sharing their data publicly. However, these data are available upon request.

## Supplemental Information

Document S1. Figures S1–S4 and Tables S1 and S2

Document S2. Article plus Supplemental Information

## References

[1] Simanova I., Hagoort P., Oostenveld R., van Gerven M.A.J. (2014). Modality-independent decoding of semantic information from the human brain. Cereb. Cortex.

[2] Fairhall S.L., Caramazza A. (2013). Brain regions that represent amodal conceptual knowledge. J. Neurosci..

[3] Shinkareva S.V., Malave V.L., Mason R.A., Mitchell T.M., Just M.A. (2011). Commonality of neural representations of words and pictures. Neuroimage.

[4] Anthony J.L., Francis D.J. (2005). Development of phonological awareness. Curr. Dir. Psychol. Sci..

[5] Araújo S., Fernandes T., Huettig F. (2019). Learning to read facilitates the retrieval of phonological representations in rapid automatized naming: evidence from unschooled illiterate, ex-illiterate, and schooled literate adults. Dev. Sci..

[6] Paivio A. (1991). Dual coding theory: retrospect and current status. Can. J. Psychol..

[7] Correia J., Formisano E., Valente G., Hausfeld L., Jansma B., Bonte M. (2014). Brain-based translation: fMRI decoding of spoken words in bilinguals reveals language-independent semantic representations in anterior temporal lobe. J. Neurosci..

[8] Buchweitz A., Shinkareva S.V., Mason R.A., Mitchell T.M., Just M.A. (2012). Identifying bilingual semantic neural representations across languages. Brain Lang..

[9] Devereux B.J., Tyler L.K., Geertzen J., Randall B. (2014). The Centre for Speech, Language and the Brain (CSLB) concept property norms. Behav. Res. Methods.

[10] MacSweeney M., Woll B., Campbell R., Calvert G.A., McGuire P.K., David A.S., Simmons A., Brammer M.J. (2002). Neural correlates of British sign language comprehension: spatial processing demands of topographic language. J. Cogn. Neurosci..

[11] Petitto L.A., Zatorre R.J., Gauna K., Nikelski E.J., Dostie D., Evans A.C. (2000). Speech-like cerebral activity in profoundly deaf people processing signed languages: implications for the neural basis of human language. Proc. Natl. Acad. Sci. USA.

[12] Emmorey K., McCullough S., Weisberg J. (2015). Neural correlates of fingerspelling, text, and sign processing in deaf American Sign Language–English bilinguals. Lang. Cogn. Neurosci..

[13] Sakai K.L., Tatsuno Y., Suzuki K., Kimura H., Ichida Y. (2005). Sign and speech: amodal commonality in left hemisphere dominance for comprehension of sentences. Brain.

[14] Söderfeldt B., Rönnberg J., Risberg J. (1994). Regional cerebral blood flow in sign language users. Brain Lang..

[15] Ralph M.A., Jefferies E., Patterson K., Rogers T.T. (2017). The neural and computational bases of semantic cognition. Nat. Rev. Neurosci..

[16] Połczyńska M.M., Japardi K., Bookheimer S.Y. (2017). Lateralizing language function with pre-operative functional magnetic resonance imaging in early proficient bilingual patients. Brain Lang..

[17] Newman A.J., Bavelier D., Corina D., Jezzard P., Neville H.J. (2002). A critical period for right hemisphere recruitment in American Sign Language processing. Nat. Neurosci..

[18] Price C.J., Wise R.J.S., Frackowiak R.S.J. (1996). Demonstrating the implicit processing of visually presented words and pseudowords. Cereb. Cortex.

[19] Vitello S., Warren J.E., Devlin J.T., Rodd J.M. (2014). Roles of frontal and temporal regions in reinterpreting semantically ambiguous sentences. Front. Hum. Neurosci..

[20] MacSweeney M., Woll B., Campbell R., McGuire P.K., David A.S., Williams S.C.R., Suckling J., Calvert G.A., Brammer M.J. (2002). Neural systems underlying British Sign Language and audio-visual English processing in native users. Brain.

[21] MacSweeney M., Campbell R., Woll B., Giampietro V., David A.S., McGuire P.K., Calvert G.A., Brammer M.J. (2004). Dissociating linguistic and nonlinguistic gestural communication in the brain. Neuroimage.

[22] MacSweeney M., Campbell R., Woll B., Brammer M.J., Giampietro V., David A.S., Calvert G.A., McGuire P.K. (2006). Lexical and sentential processing in British Sign Language. Hum. Brain Mapp..

[23] Sapir E. (1929). The status of linguistics as a science. Language.

[24] Whorf B.L., Carroll J.B. (1956). Language, Thought and Reality: Selected Writings of Benjamin Lee Whorf.

[25] Van de Putte E., De Baene W., Price C.J., Duyck W. (2018). Neural overlap of L1 and L2 semantic representations across visual and auditory modalities: a decoding approach. Neuropsychologia.

[26] de Groot A.M. (1992). Determinants of word translation. J. Exp. Psychol. Learn. Mem. Cogn..

[27] Duyck W., Brysbaert M. (2004). Forward and backward number translation requires conceptual mediation in both balanced and unbalanced bilinguals. J. Exp. Psychol. Hum. Percept. Perform..

[28] Van Hell J.G., De Groot A.M.B. (1998). Conceptual representation in bilingual memory: Effects of concreteness and cognate status in word association. Biling. Lang. Cogn..

[29] Zwitserlood I., Kristoffersen J., Troelsgard T., Jackson H. (2013). Issues in sign language lexicography. The Bloomsbury Campanion to Lexicography.

[30] Marslen-Wilson W., Altmann G.T.M. (1995). Activation, competition, and frequency in lexical access. Cognitive Models of Speech Processing: Psycholinguistic and Computational Perspectives.

[31] Apfelbaum K.S., Blumstein S.E., McMurray B. (2011). Semantic priming is affected by real-time phonological competition: evidence for continuous cascading systems. Psychon. Bull. Rev..

[32] Perniss P., Thompson R.L., Vigliocco G. (2010). Iconicity as a general property of language: evidence from spoken and signed languages. Front. Psychol..

[33] Marshall C., Rowley K., Atkinson J. (2014). Modality-dependent and -independent factors in the organisation of the signed language lexicon: insights from semantic and phonological fluency tasks in BSL. J. Psycholinguist. Res..

[34] Padden C.A., Meir I., Hwang S.O., Lepic R., Seegers S., Sampson T. (2013). Patterned iconicity in sign language lexicons. Gesture.

[35] MacSweeney M., Capek C.M., Campbell R., Woll B. (2008). The signing brain: the neurobiology of sign language. Trends Cogn. Sci..

[36] Nili H., Wingfield C., Walther A., Su L., Marslen-Wilson W., Kriegeskorte N. (2014). A toolbox for representational similarity analysis. PLoS Comput. Biol..

[37] Davis C.J. (2005). N-watch: a program for deriving neighborhood size and other psycholinguistic statistics. Behav. Res. Methods.

[38] Kuperman V., Stadthagen-Gonzalez H., Brysbaert M. (2012). Age-of-acquisition ratings for 30,000 English words. Behav. Res. Methods.

[39] Wilson M. (1988). MRC psycholinguistic database: machine usable dictionary, version 2.00. Behav. Res. Methods.

[40] Vinson D.P., Cormier K., Denmark T., Schembri A., Vigliocco G. (2008). The British Sign Language (BSL) norms for age of acquisition, familiarity, and iconicity. Behav. Res. Methods.

[41] Levenshtein V.I. (1966). Binary codes capable of correcting deletions, insertions, and reversals. Sov. Phys. Dokl..

[42] Fenlon J., Cormier K., Schembri A. (2015). Building BSL SignBank: the lemma dilemma revisited. Int. J. Lexicogr..

[43] Kriegeskorte N., Mur M. (2012). Inverse MDS: inferring dissimilarity structure from multiple item arrangements. Front. Psychol..

[44] Walther A., Nili H., Ejaz N., Alink A., Kriegeskorte N., Diedrichsen J. (2016). Reliability of dissimilarity measures for multi-voxel pattern analysis. Neuroimage.

[45] Kriegeskorte N., Goebel R., Bandettini P. (2006). Information-based functional brain mapping. Proc. Natl. Acad. Sci. USA.

[46] Evans S., Davis M.H. (2015). Hierarchical organization of auditory and motor representations in speech perception: Evidence from searchlight similarity analysis. Cereb. Cortex.

[47] Poldrack R.A., Baker C.I., Durnez J., Gorgolewski K.J., Matthews P.M., Munafò M.R., Nichols T.E., Poline J.B., Vul E., Yarkoni T. (2017). Scanning the horizon: towards transparent and reproducible neuroimaging research. Nat. Rev. Neurosci..

[48] Friston K.J., Rotshtein P., Geng J.J., Sterzer P., Henson R.N. (2006). A critique of functional localisers. Neuroimage.

[49] Walker D.A. (2003). JMASM9: converting Kendall’s tau for correlational or meta-analytic analyses. J. Mod. Appl. Stat. Methods.

[50] Kaplan J.T., Man K., Greening S.G. (2015). Multivariate cross-classification: applying machine learning techniques to characterize abstraction in neural representations. Front. Hum. Neurosci..

[51] Kriegeskorte N., Simmons W.K., Bellgowan P.S., Baker C.I. (2009). Circular analysis in systems neuroscience: the dangers of double dipping. Nat. Neurosci..

[52] Diedrichsen J., Kriegeskorte N. (2017). Representational models: a common framework for understanding encoding, pattern-component, and representational-similarity analysis. PLoS Comput. Biol..

[53] Esterman M., Tamber-Rosenau B.J., Chiu Y.C., Yantis S. (2010). Avoiding non-independence in fMRI data analysis: leave one subject out. Neuroimage.

