# Supplementary material for: Sign and Speech Share Partially Overlapping Conceptual Representations
Source: Curr Biol. 2019 Nov 4;29(21):3739–3747.e5. doi: 10.1016/j.cub.2019.08.075 (PMC6839399; doi:10.1016/j.cub.2019.08.075)
Supplement: Document S1. Figures S1–S4 and Tables S1 and S2 [file mmc1.pdf]

Current Biology, Volume *29*

Supplemental Information

Sign and Speech Share Partially Overlapping Conceptual Representations

Samuel Evans, Cathy J. Price, Jörn Diedrichsen, Eva Gutierrez-Sigut, and Mairéad MacSweeney

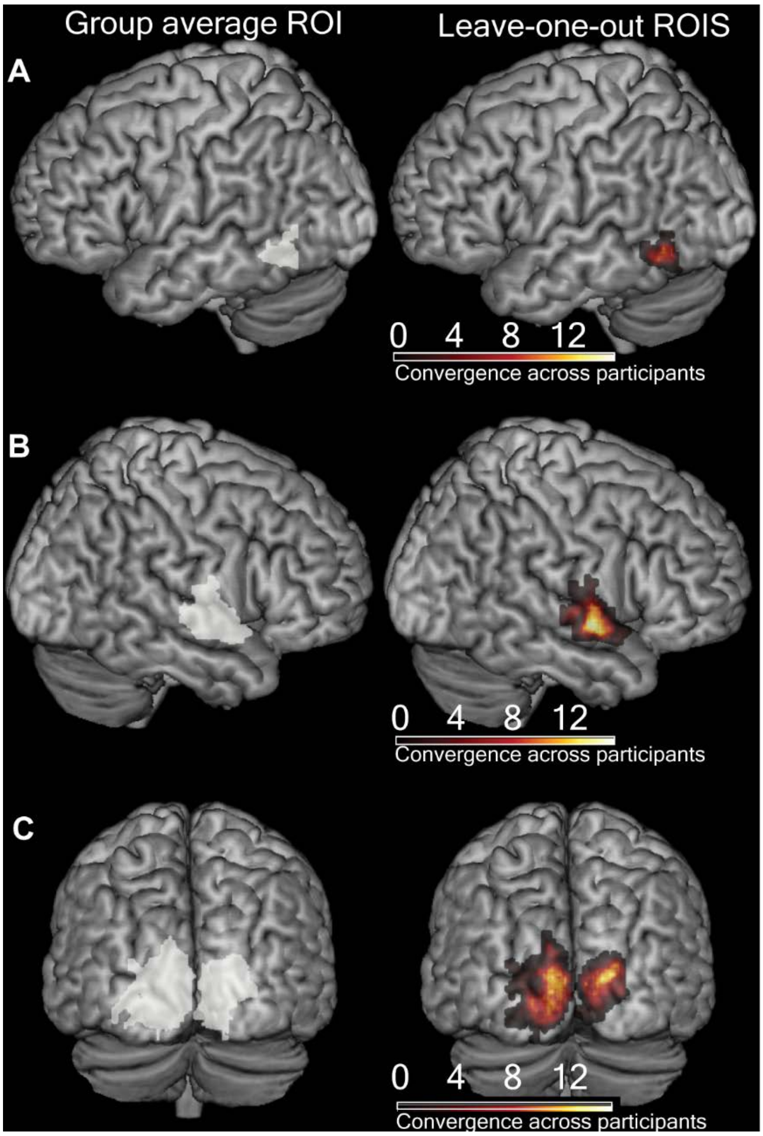

Figure S1: Definition of leave-one-participant-out regions of Interest, Related to STAR Methods, Figure 2, Figure 3 and Figure 4

A leave-one-participant-out procedure [S1] was used to test for speaker and signer identity models to ensure that the evaluation of the model was statistically independent of the process used to generate the ROIs. Rendered with MRICRON on the Ch2better brain.

(A-C) Left side panels show the group average ROI using the data from all participants. Right side panels show the overlap of the leave-one-participant-out ROIs across participants.

(A) ROI in the left pMTG/ITG [-48 -62 -6] generated by a searchlight analysis testing for > 0 within modality distances and associated leave-one-participant-out ROIs.

(B) ROI in the right anterior STG [58 -4 -2] generated by a searchlight analysis testing for speech > sign distances and associated leave-one-participant-out ROIs.

(C) ROIs in the left V1-V3 [-6 -98 16] and the right V1-V3 [22 -90 16] generated by a searchlight analysis testing for sign > speech distances and associated leave-one-participant-out ROIs.

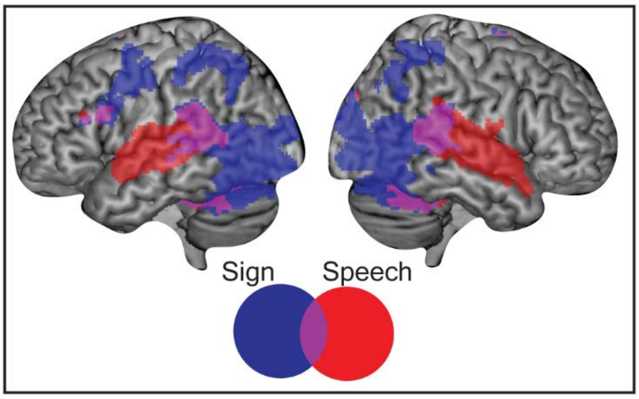

Figure S2: Univariate overlap between sign and speech, Related to Figure 2

Areas responding to speech (red) and sign (blue) compared to rest and their overlap (pink), thresholded at $p < 0.005$ peak level, $q < 0.05$ FDR corrected at the cluster level. Rendered with MRICRON on the Ch2better brain. As expected, areas of shared univariate activity for sign and speech were found in the bilateral posterior superior and the middle temporal gyrus, the left inferior frontal gyrus and bilateral cerebellum.

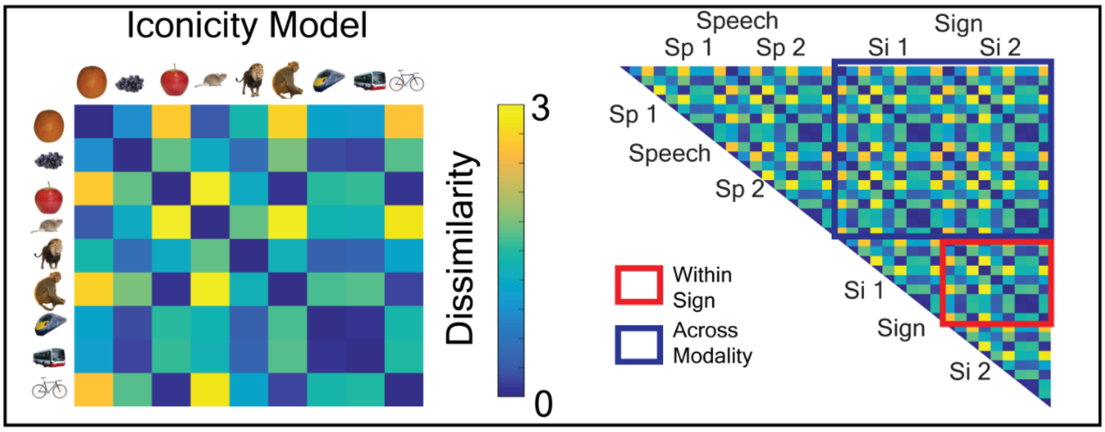

Figure S3: Effects of iconicity, Related to Figure 2

An iconicity model (left) was derived from the group average iconicity ratings for each sign. This model was created by taking the absolute value from the subtraction of the average iconicity value of each sign from every other sign. The model was tested (right) on the sign-sign distances (red box), e.g. within sign, and the speech-sign distances (blue box), e.g. across-modality. There was no significant fit to the within sign (t (16) = 0.382, p = 0.354, d_z = 0.093) or across-modality distances (t (16) = 1.298, p = 0.106, d_z = 0.315) in the left pMTG/ITG. An additional iconicity model was tested that used each individuals' iconicity ratings for each exemplar of each sign. As with the model using the group averaged iconicity values, there was no significant fit in the within sign (t (16) = 0.588, p = 0.282, dz = 0.143) or across-modality distances (t (16) = 0.277, p = 0.393, dz = 0.067).

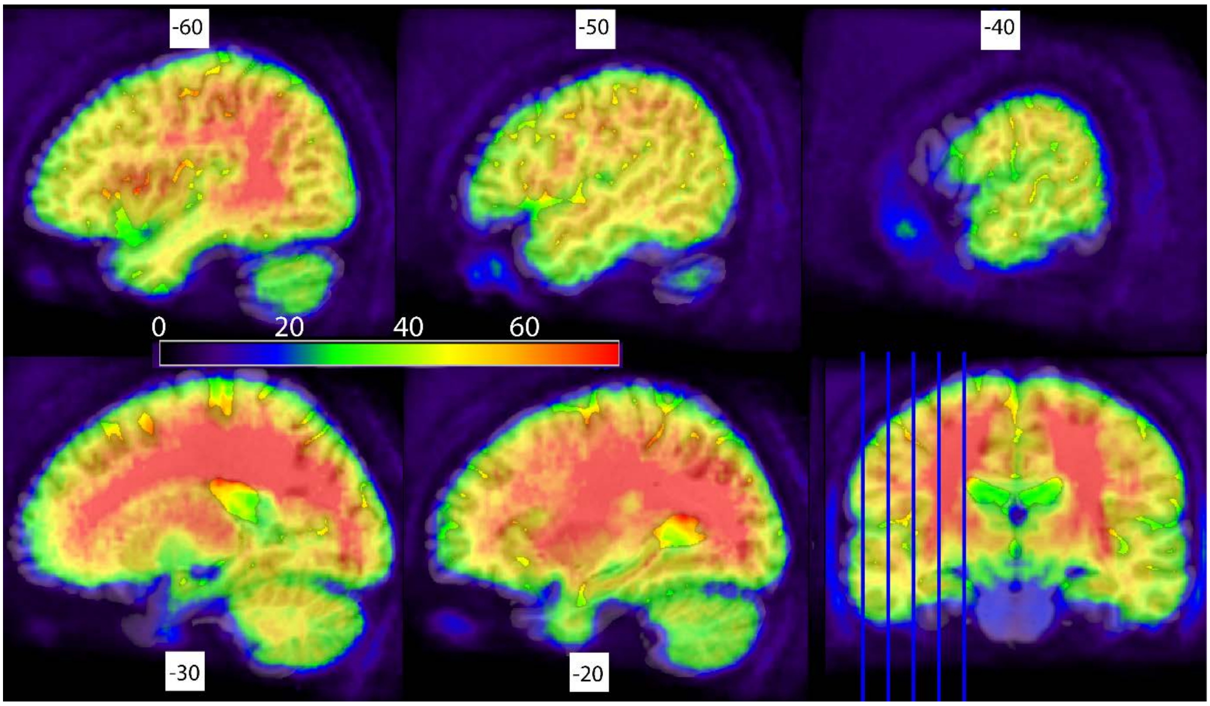

Figure S4: Signal quality in the temporal lobe, Related to Figure 2

Whole brain tSNR maps for the group. Sagittal slices of the left temporal lobe are shown. The mid-anterior temporal lobe has been ascribed an important role in ‘amodal’ semantic cognition. Within this area, a gradient of function from posterior-anterior has been suggested that reflects a wider-to-narrower window of semantic specificity, e.g. from categories to items and individual exemplars [S2,S3]. This region is particularly susceptible to signal drop out [S4,S5]. However, tSNR maps indicated relatively good signal quality in the mid-anterior inferior temporal cortex and drop out that was similar to that found in the left pMTG/ITG. We chose not to use a dual echo sequence to mitigate against drop out, as our sequence was optimised for signal quality in the posterior temporal cortex, the region most consistently activated by both sign and speech in previous univariate studies. The absence of shared item-level correspondences might also reflect the fact that participants were asked to monitor for category rather than item-level distinctions [S6]. We decided to use a category-based task to maximise the likelihood of finding commonality between the languages, which we assumed would be more robust at a broader level of semantic specificity. Future studies using dual echo sequences and item-level discriminative tasks are necessary to exclude the possibility that these methodological details obscured identification of item-level correspondences.

Item	Age of acquisition	Imageability	Familiarity	Syllables	Phonemes	Iconicity
Orange	3.26	626	567	2	5	2.56
Grapes	3.94[a]	591[a]	532[a]	1	5	3.50
Apple	4.15	637	598	2	3	5.35
**Mean**	**3.78**	**618**	**566**	**2**	**4**	**3.80**
Mouse	4.94	615	520	1	3	2.24
Lion	4.42	626	511	2	4	4.09
Monkey	4.21	588	531	2	5	5.44
**Mean**	**4.52**	**610**	**521**	**2**	**4**	**3.92**
Train	4	593	548	1	4	**3.74**
Bus	3.85	624[c]	513[c]	1	3	**3.68**
Bicycle	4.26	649[b]	591[b]	3	6	**5.26**
**Mean**	**4.04**	**622**	**551**	**2**	**4**	**4.23**

Table S1: The psycholinguistic properties of the core items, Related to STAR Methods.

Imageability (Bristol/MRC), subjective familiarity (MRC database), number of syllables and phonemes extracted from the N-Watch program [S7], age of acquisition was extracted from Kuperman et al. [S8] and iconicity values were acquired directly from the participants. Note that [a]the term “grape” was used in the absence of the term “grapes” for age of acquisition, familiarity and imageability ratings, [b]the term “bike” was used in the absence of the term “bicycle” for familiarity and imageability ratings and [c]the term coach was used in the absence of “bus” for familiarity and imageability ratings.

Region	x	y	z	Extent	Z Value
Within-modality representational structure					
Right superior temporal gyrus	58	-4	-2	1545	5.283
Right inferior parietal lobule	64	-30	14		4.968
Right superior temporal gyrus	52	-2	-8		4.861
Left superior occipital gyrus	-14	-96	10	2629	4.677
Right superior occipital gyrus	14	-100	16		4.479
Right cuneus	6	-92	22		4.226
Left superior temporal gyrus	-60	-10	-2	1276	4.500
Left middle temporal gyrus	-64	-30	6		4.476
Left middle temporal gyrus	-64	-44	2		4.175
Left inferior temporal gyrus	-48	-62	-6	172	4.361
Left middle occipital gyrus	-42	-64	0		3.122
Right insula	36	-12	14	194	4.178
Right putamen	30	-8	10		4.160
Right middle temporal gyrus	52	-68	6	279	3.954
Right middle temporal gyrus	56	-48	0		3.748
Right middle temporal gyrus	54	-54	6		3.574
Speech > Sign					
Right superior temporal gyrus	58	-4	-2	754	4.877
Right superior temporal gyrus	52	0	-8		4.779
Right superior temporal gyrus	60	-12	4		3.590
Left superior temporal gyrus	-56	-8	2	743	4.484
Left superior temporal gyrus	-62	-30	10		4.253
Left superior temporal gyrus	-62	-2	0		3.720

Right Putamen	30	-10	10	146	4.364
Right Insular	40	-12	10		3.354
Right superior temporal gyrus	58	-34	18	285	4.160
Right superior temporal gyrus	66	-32	14		3.763
Right superior temporal gyrus	56	-26	0		3.722
Sign > Speech					
Left cuneus	-6	-98	16	1145	4.623
Left middle occipital gyrus	-12	-102	4		4.019
Left cuneus	-8	-94	28		3.830
Right superior occipital gyrus	22	-90	16	969	4.375
Right lingual gyrus	16	-84	-4		3.976
Right cuneus	16	-100	12		3.655
Left inferior occipital gyrus	-44	-80	-6	264	4.107
Left middle occipital gyrus	-50	-72	-2		3.937
Left middle occipital gyrus	-42	-80	4		3.449
Left cerebellum	-4	-48	-8	116	3.808
Left lingual gyrus	-10	-56	-2		3.767
Left cerebellum	-4	-50	0		3.102
Left superior occipital gyrus	-10	-84	42	127	3.781
Left superior occipital gyrus	-16	-78	40		3.396
Left superior parietal lobule	-26	-80	48		3.172

Table S2: **MNI coordinates for RSA searchlight analyses, Related to Figure 2, Figure 3 and Figure 4.**

3 local maxima more than 8 mm apart

SUPPLEMENTAL REFERENCES

S1. Esterman, M., Tamber-Rosenau, B.J., Chiu, Y.-C., and Yantis, S. (2010). Avoiding non-independence in fMRI data analysis: leave one subject out. Neuroimage *50*, 572–6. Available at: http://www.pubmedcentral.nih.gov/articlerender.fcgi?artid=2823971&tool=pmcentrez&rendertype=abstract [Accessed September 22, 2014].

S2. Lambon Ralph, M., Jefferies, E., Patterson, K., and Rogers, T.T. (2016). The neural and computational bases of semantic cognition. Nat. Rev. Neurosci. *18*, 42–55. Available at: http://dx.doi.org/10.1038/nrn.2016.150.

S3. Clarke, A., and Tyler, L.K. (2015). Understanding What We See: How We Derive Meaning From Vision. Trends Cogn. Sci. *19*, 677–687. Available at: http://dx.doi.org/10.1016/j.tics.2015.08.008.

S4. Devlin, J.T., Russell, R.P., Davis, M.H., Price, C.J., Wilson, J., Moss, H.E., Matthews, P.M., and Tyler, L.K. (2000). Susceptibility-induced loss of signal: Comparing PET and fMRI on a semantic task. Neuroimage *11*, 589–600. Available at: isi:000087963600002.

S5. Halai, A.D., Welbourne, S.R., Embleton, K., and Parkes, L.M. (2014). A comparison of dual gradient-echo and spin-echo fMRI of the inferior temporal lobe. Hum. Brain Mapp. *35*, 4118–28. Available at: http://www.ncbi.nlm.nih.gov/pubmed/24677506 [Accessed July 22, 2015].

S6. Bonte, M., Hausfeld, L., Scharke, W., Valente, G., and Formisano, E. (2014). Task-dependent decoding of speaker and vowel identity from auditory cortical

response patterns. J. Neurosci. *34*, 4548–57. Available at: http://www.ncbi.nlm.nih.gov/pubmed/24672000 [Accessed June 3, 2014].

S7. Davis, C.J. (2005). N-Watch: A program for deriving neighbourhood size and other pyscholinguistic statistics. Behav. Res. Methods *37*, 65–70.

S8. Kuperman, V., Stadthagen-Gonzalez, H., and Brysbaert, M. (2012). Age-of-acquisition ratings for 30,000 English words. Behav. Res. Methods *44*, 978–990.
